# *catena*-Poly[[bis­(*N*,*N*′-dimethyl­formamide)cadmium(II)]-μ_2_-oxalato]

**DOI:** 10.1107/S1600536807066147

**Published:** 2007-12-12

**Authors:** Cédric Borel, Vratislav Langer, Johan Arnehed, Lisette Leikvoll, Mohamed Ghazzali

**Affiliations:** a Department of Chemical and Biological Engineering, Chalmers University of Technology, 41296 Göteborg, Sweden; b Chemistry Department, Faculty of Science, Alexandria University, PO Box 426, 21321 Alexandria, Egypt

## Abstract

The title compound, [Cd(C_2_O_4_)(C_3_H_7_NO)_2_]_*n*_, is isostructural with its Mn^II^ analogue. The structure comprises zigzag polymeric chains with the oxalate groups situated on inversion centres and the Cd^II^ atoms located on twofold rotation axes. The coordination geometry around Cd^II^ is distorted octa­hedral and the intra­chain Cd⋯Cd distance is 5.842 (1) Å. C—H⋯O hydrogen bonds exist between the parallel polymeric chains.

## Related literature

For the isostructural Mn^II^ analogue, see: Chan *et al.* (2007 ▶). For related literature, see: Borel *et al.* (2006 ▶); Decurtins *et al.* (1994 ▶); Imaz *et al.* (2005 ▶); Ma *et al.* (2007 ▶); Ockwig *et al.* (2005 ▶); Prasad *et al.* (2002 ▶); Xia *et al.* (2004 ▶); Zavalij *et al.* (2003 ▶); Zaworotko (2007 ▶).
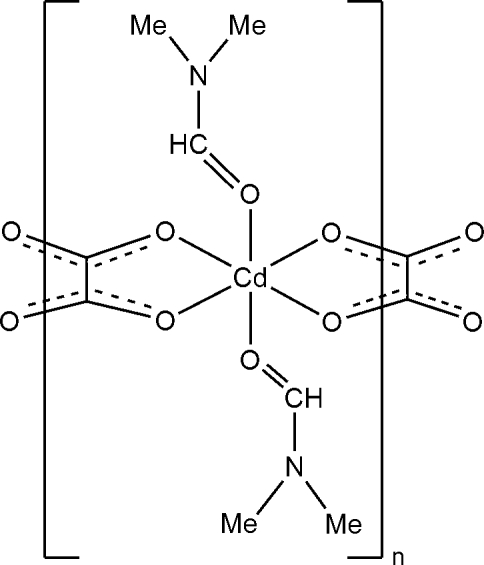


## Experimental

### 

#### Crystal data

[Cd(C_2_O_4_)(C_3_H_7_NO)_2_]
*M* *_r_* = 346.61
Orthorhombic, 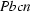

*a* = 15.153 (4) Å
*b* = 8.006 (2) Å
*c* = 10.403 (3) Å
*V* = 1262.0 (6) Å^3^
*Z* = 4
Mo *K*α radiation
μ = 1.75 mm^−1^
*T* = 153 (2) K
0.41 × 0.31 × 0.19 mm

#### Data collection

Siemens SMART CCD diffractometer
Absorption correction: multi-scan (*SADABS*; Sheldrick, 2003 ▶) *T*_min_ = 0.523, *T*_max_ = 0.718
19498 measured reflections
2301 independent reflections
1705 reflections with *I* > 2σ(*I*)
*R*_int_ = 0.055

#### Refinement

*R*[*F* ^2^ > 2σ(*F* ^2^)] = 0.025
*wR*(*F* ^2^) = 0.077
*S* = 1.01
2301 reflections
80 parameters
H-atom parameters constrained
Δρ_max_ = 1.28 e Å^−3^
Δρ_min_ = −0.75 e Å^−3^

### 

Data collection: *SMART* (Bruker, 2003 ▶); cell refinement: *SAINT* (Bruker, 2003 ▶); data reduction: *SAINT*; program(s) used to solve structure: *SHELXTL* (Bruker, 2003 ▶); program(s) used to refine structure: *SHELXTL*; molecular graphics: *DIAMOND* (Brandenburg, 2007 ▶); software used to prepare material for publication: *SHELXTL*.

## Supplementary Material

Crystal structure: contains datablocks I, global. DOI: 10.1107/S1600536807066147/bi2270sup1.cif

Structure factors: contains datablocks I. DOI: 10.1107/S1600536807066147/bi2270Isup2.hkl

Additional supplementary materials:  crystallographic information; 3D view; checkCIF report

## Figures and Tables

**Table 1:** Hydrogen-bond geometry (Å, °)

*D*—H⋯*A*	*D*—H	H⋯*A*	*D*⋯*A*	*D*—H⋯*A*
C4—H4*B*⋯O1^i^	0.98	2.65	3.456 (2)	140
C4—H4*C*⋯O2^ii^	0.98	2.70	3.516 (3)	141
C4—H4*C*⋯O1^iii^	0.98	2.63	3.468 (3)	144
C4—H4*A*⋯O3	0.98	2.36	2.775 (2)	104

Symmetry codes: (i) 
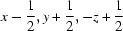
; (ii) 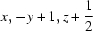; (iii) 
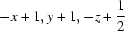
.

**Fig. 1.**
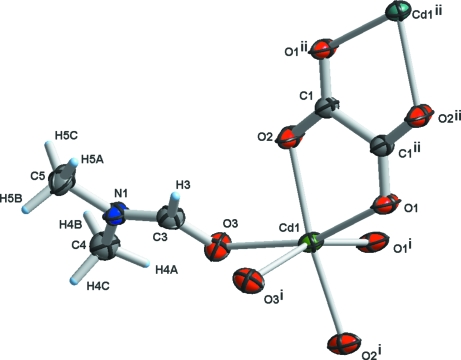
Perspective drawing showing the atom-numbering scheme and atomic displacement ellipsoids at the 50% probability level for non-H atoms. Symmetry codes: (i) -x + 1, y, -z + 1/2; (ii) -x + 1, -y, -z.

**Fig. 2.**
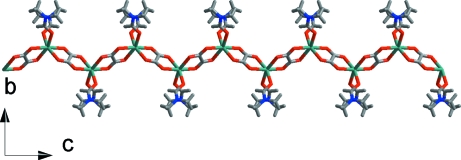
A projection in the bc-plane showing the one-dimensional chain propagating along the c-direction.

## supplementary crystallographic information

### Comment

Crystal engineering of coordination polymers, based on pre-defined interactions of metal ions with organic spacers, is an area of research that has received substantial interest (Zaworotko, 2007). In this field, employing N– and/or O– donor ligands as bridging organic modules has been intensively implemented (Ockwig *et al.*, 2005). Oxalate anions are known as chelating bis-bidentate ligands and many infinite two-dimensional and three-dimensional coordination polymers with a [*MM*'(ox)_n_]_n' _formula have been reported comprising two different and/or similar metal centres (Borel *et al.*, 2006: Imaz *et al.*, 2005: Xia *et al.*, 2004: Decurtins *et al.*, 1994). However, solvent ligation to the metal centres may result in structures with lower dimensionality (Prasad *et al.*, 2002). Here we present a coordination chain based on bis-oxalato cadmium(II) with coordinated DMF solvent molecules.

A perspective drawing of the title compound with the atomic numbering scheme is shown in Figure 1. The Cd^II^ ions are situated on crystallographic twofold rotation axes while the oxalates are located on inversion centres. The Cd^II^ ion displays a distorted octahedral coordination geometry with two dimethylformamide molecules ligated to the Cd^II^ centre and the zigzag chain is built up from two oxalate units, linked *via* four O atoms to two Cd^II^ ions with a Cd—O distance in the range 2.262 (1)–2.297 (1) Å [(Cd—O)average = 2.275 (19) Å] (Figure 2). The intrachain Cd···Cd distance is 5.842 (1) Å. Contrary to many oxalate-metal chains which are linked to each other in one direction by π-π interactions (Ma *et al.*, 2007) this structure exhibits only C—H···O hydrogen bonds which are both interchain and intrachain. The intermolecular hydrogen bonds build a stack of chains with a Cd···Cd distance of 8.006 (2) Å in the *b* axis direction and 8.569 (2) Å in the *a* axis direction. The three-dimensional architecture is maintained *via* coordination/covalent bonding in the *c*-direction and weaker C—H···O intermolecular hydrogen bonds in the *ab*-plane.

### Experimental

All chemicals used in the first step of the synthesis were purchased from Aldrich and used without further purification. 1.81 g (2 mmol) oxalic acid was dissolved in 15 ml H_2_O. 0.42 g (1 mmol) LiOH.H_2_O and 0.62 g (1 mmol) H_3_BO_3_ were dissolved in 15 ml H_2_O and added to the solution.The mixture was brought to boiling and evaporated to dryness. The resulting Li[B(ox)_2_] was dried in a desiccator (Zavalij *et al.*, 2003). A solution of 3.9 g Li[B(ox)_2_] in 50 ml DMF was prepared and heated to 343 K. A precipitate formed, probably a sign of the disintegration of the bis(oxalate)borate ion, and the solution was filtered. One eighth of this filtrate was then mixed with a solution of 0.2 g C d(NO_3_)_2_.4H_2_O and the resulting solution was set aside for 1–2 weeks, after which colourless prismatic crystals suitable for *x*-ray diffraction were collected and dried.

### Refinement

H atoms were placed in idealized positions and refined using a riding model with *U*_iso_(H) = 1.2 *U*_eq_(C).

### Crystal data

**Table:**

[Cd(C_2_O_4_)(C_3_H_7_NO)_2_]	*F*_000_ = 688
*M**_r_* = 346.61	*D*_x_ = 1.824 Mg m^−^^3^
Orthorhombic, *P**b**c**n*	Mo *K*α radiation λ = 0.71073 Å
Hall symbol: -P 2n 2ab	Cell parameters from 2301 reflections
*a* = 15.153 (4) Å	θ = 2.7–32.9º
*b* = 8.006 (2) Å	µ = 1.75 mm^−^^1^
*c* = 10.403 (3) Å	*T* = 153 (2) K
*V* = 1262.0 (6) Å^3^	Prism, colourless
*Z* = 4	0.41 × 0.31 × 0.19 mm

### Data collection

**Table:**

Siemens SMART CCD diffractometer	2301 independent reflections
Radiation source: fine-focus sealed tube	1705 reflections with *I* > 2σ(*I*)
Monochromator: graphite	*R*_int_ = 0.055
*T* = 153(2) K	θ_max_ = 32.9º
ω scans	θ_min_ = 2.7º
Absorption correction: multi-scan(SADABS; Sheldrick, 2003)	*h* = −23→23
*T*_min_ = 0.523, *T*_max_ = 0.718	*k* = −12→12
19498 measured reflections	*l* = −15→15

### Refinement

**Table:**

Refinement on *F*^2^	Secondary atom site location: difference Fourier map
Least-squares matrix: full	Hydrogen site location: inferred from neighbouring sites
*R*[*F*^2^ > 2σ(*F*^2^)] = 0.025	H-atom parameters constrained
*wR*(*F*^2^) = 0.077	*w* = 1/[σ^2^(*F*_o_^2^) + (0.0446*P*)^2^ + 0.4422*P*] where *P* = (*F*_o_^2^ + 2*F*_c_^2^)/3
*S* = 1.01	(Δ/σ)_max_ < 0.001
2301 reflections	Δρ_max_ = 1.28 e Å^−^^3^
80 parameters	Δρ_min_ = −0.75 e Å^−^^3^
Primary atom site location: structure-invariant direct methods	Extinction correction: none

### Special details

**Table:**

Geometry. All e.s.d.'s (except the e.s.d. in the dihedral angle between two l.s. planes) are estimated using the full covariance matrix. The cell e.s.d.'s are taken into account individually in the estimation of e.s.d.'s in distances, angles and torsion angles; correlations between e.s.d.'s in cell parameters are only used when they are defined by crystal symmetry. An approximate (isotropic) treatment of cell e.s.d.'s is used for estimating e.s.d.'s involving l.s. planes.
Refinement. Refinement of *F*^2^ against ALL reflections. The weighted *R*-factor *wR* and goodness of fit *S* are based on *F*^2^, conventional *R*-factors *R* are based on *F*, with *F* set to zero for negative *F*^2^. The threshold expression of *F*^2^ > σ(*F*^2^) is used only for calculating *R*-factors(gt) *etc*. and is not relevant to the choice of reflections for refinement. *R*-factors based on *F*^2^ are statistically about twice as large as those based on *F*, and *R*- factors based on ALL data will be even larger.

### Fractional atomic coordinates and isotropic or equivalent isotropic displacement parameters (Å^2^)

**Table:**

	*x*	*y*	*z*	*U*_iso_*/*U*_eq_	
Cd1	0.5000	0.16606 (2)	0.2500	0.01965 (7)	
O2	0.42585 (9)	0.14921 (17)	0.06167 (13)	0.0290 (3)	
O1	0.57439 (8)	−0.02193 (18)	0.12849 (12)	0.0285 (3)	
O3	0.40504 (9)	0.37996 (18)	0.30029 (13)	0.0278 (3)	
N1	0.33333 (10)	0.6091 (2)	0.22806 (14)	0.0234 (3)	
C3	0.38865 (12)	0.4853 (2)	0.21484 (18)	0.0247 (3)	
H3	0.4185	0.4745	0.1349	0.030*	
C1	0.45726 (11)	0.0493 (2)	−0.01929 (16)	0.0212 (3)	
C5	0.31781 (15)	0.7309 (3)	0.1264 (2)	0.0377 (5)	
H5A	0.3549	0.7041	0.0521	0.057*	
H5B	0.3325	0.8429	0.1579	0.057*	
H5C	0.2556	0.7277	0.1010	0.057*	
C4	0.28609 (13)	0.6360 (3)	0.34853 (19)	0.0291 (4)	
H4A	0.2970	0.5418	0.4066	0.044*	
H4B	0.2227	0.6445	0.3312	0.044*	
H4C	0.3068	0.7395	0.3887	0.044*	

### Atomic displacement parameters (Å^2^)

**Table:**

	*U*^11^	*U*^22^	*U*^33^	*U*^12^	*U*^13^	*U*^23^
Cd1	0.02016 (11)	0.02378 (11)	0.01502 (10)	0.000	−0.00112 (5)	0.000
O2	0.0281 (6)	0.0385 (7)	0.0203 (6)	0.0124 (5)	−0.0054 (5)	−0.0067 (5)
O1	0.0272 (6)	0.0388 (8)	0.0194 (5)	0.0088 (5)	−0.0083 (4)	−0.0069 (5)
O3	0.0297 (7)	0.0312 (7)	0.0224 (6)	0.0071 (6)	0.0048 (5)	0.0032 (6)
N1	0.0242 (7)	0.0274 (8)	0.0187 (6)	0.0024 (6)	−0.0015 (5)	−0.0004 (6)
C3	0.0259 (8)	0.0293 (9)	0.0189 (7)	0.0019 (7)	0.0027 (6)	−0.0014 (7)
C1	0.0197 (8)	0.0251 (7)	0.0186 (7)	0.0027 (6)	−0.0025 (5)	−0.0002 (6)
C5	0.0463 (12)	0.0414 (12)	0.0254 (9)	0.0106 (10)	−0.0008 (8)	0.0063 (9)
C4	0.0246 (9)	0.0371 (10)	0.0257 (9)	0.0038 (7)	0.0044 (7)	−0.0024 (8)

### Geometric parameters (Å, °)

**Table:**

Cd1—O2^i^	2.2624 (14)	N1—C4	1.459 (2)
Cd1—O2	2.2624 (14)	C3—H3	0.9500
Cd1—O1^i^	2.2658 (13)	C1—O1^ii^	1.2524 (19)
Cd1—O1	2.2658 (13)	C1—C1^ii^	1.569 (3)
Cd1—O3	2.2971 (14)	C5—H5A	0.9800
Cd1—O3^i^	2.2972 (14)	C5—H5B	0.9800
O2—C1	1.255 (2)	C5—H5C	0.9800
O1—C1^ii^	1.2524 (19)	C4—H4A	0.9800
O3—C3	1.250 (2)	C4—H4B	0.9800
N1—C3	1.305 (2)	C4—H4C	0.9800
N1—C5	1.457 (3)		
			
O2^i^—Cd1—O2	173.16 (7)	C5—N1—C4	116.45 (17)
O2^i^—Cd1—O1^i^	74.00 (5)	O3—C3—N1	124.40 (18)
O2—Cd1—O1^i^	101.33 (5)	O3—C3—H3	117.8
O2^i^—Cd1—O1	101.33 (5)	N1—C3—H3	117.8
O2—Cd1—O1	74.00 (5)	O1^ii^—C1—O2	125.09 (16)
O1^i^—Cd1—O1	96.75 (8)	O1^ii^—C1—C1^ii^	117.39 (18)
O2^i^—Cd1—O3	99.11 (5)	O2—C1—C1^ii^	117.52 (17)
O2—Cd1—O3	86.02 (5)	N1—C5—H5A	109.5
O1^i^—Cd1—O3	93.24 (5)	N1—C5—H5B	109.5
O1—Cd1—O3	159.05 (5)	H5A—C5—H5B	109.5
O2^i^—Cd1—O3^i^	86.02 (5)	N1—C5—H5C	109.5
O2—Cd1—O3^i^	99.11 (5)	H5A—C5—H5C	109.5
O1^i^—Cd1—O3^i^	159.05 (5)	H5B—C5—H5C	109.5
O1—Cd1—O3^i^	93.24 (5)	N1—C4—H4A	109.5
O3—Cd1—O3^i^	83.60 (7)	N1—C4—H4B	109.5
C1—O2—Cd1	115.51 (11)	H4A—C4—H4B	109.5
C1^ii^—O1—Cd1	115.58 (11)	N1—C4—H4C	109.5
C3—O3—Cd1	117.74 (12)	H4A—C4—H4C	109.5
C3—N1—C5	122.37 (16)	H4B—C4—H4C	109.5
C3—N1—C4	121.15 (17)		
			
O1^i^—Cd1—O2—C1	−93.56 (14)	O2—Cd1—O3—C3	−43.38 (14)
O1—Cd1—O2—C1	0.29 (13)	O1^i^—Cd1—O3—C3	−144.53 (14)
O3—Cd1—O2—C1	173.92 (14)	O1—Cd1—O3—C3	−26.0 (2)
O3^i^—Cd1—O2—C1	91.07 (14)	O3^i^—Cd1—O3—C3	56.26 (12)
O2^i^—Cd1—O1—C1^ii^	174.51 (13)	Cd1—O3—C3—N1	177.06 (15)
O2—Cd1—O1—C1^ii^	−0.35 (13)	C5—N1—C3—O3	178.7 (2)
O1^i^—Cd1—O1—C1^ii^	99.55 (14)	C4—N1—C3—O3	1.1 (3)
O3—Cd1—O1—C1^ii^	−18.4 (2)	Cd1—O2—C1—O1^ii^	179.68 (15)
O3^i^—Cd1—O1—C1^ii^	−98.90 (14)	Cd1—O2—C1—C1^ii^	−0.2 (3)
O2^i^—Cd1—O3—C3	141.17 (14)		

Symmetry codes: (i) −*x*+1, *y*, −*z*+1/2; (ii) −*x*+1, −*y*, −*z*.

### Hydrogen-bond geometry (Å, °)

**Table:**

*D*—H···*A*	*D*—H	H···*A*	*D*···*A*	*D*—H···*A*
C4—H4B···O1^iii^	0.98	2.65	3.456 (2)	140
C4—H4C···O2^iv^	0.98	2.70	3.516 (3)	141
C4—H4C···O1^v^	0.98	2.63	3.468 (3)	144
C4—H4A···O3	0.98	2.36	2.775 (2)	104

Symmetry codes: (iii) *x*−1/2, *y*+1/2, −*z*+1/2; (iv) *x*, −*y*+1, *z*+1/2; (v) −*x*+1, *y*+1, −*z*+1/2.
